# Microfluidic Controlled Self-Assembly of Polylactide (PLA)-Based Linear and Graft Copolymers into Nanoparticles with Diverse Morphologies

**DOI:** 10.1021/acspolymersau.4c00033

**Published:** 2024-05-31

**Authors:** Svetlana Lukáš Petrova, Vladimir Sincari, Ewa Pavlova, Václav Pokorný, Volodymyr Lobaz, Martin Hrubý

**Affiliations:** Institute of Macromolecular Chemistry v.v.i., Academy of Sciences of the Czech Republic, Heyrovsky, Sq. 2, 162 06 Prague 6, Czech Republic

**Keywords:** polylactide (PLA)-based copolymers, microfluidic, micelles, polymersomes and worms

## Abstract

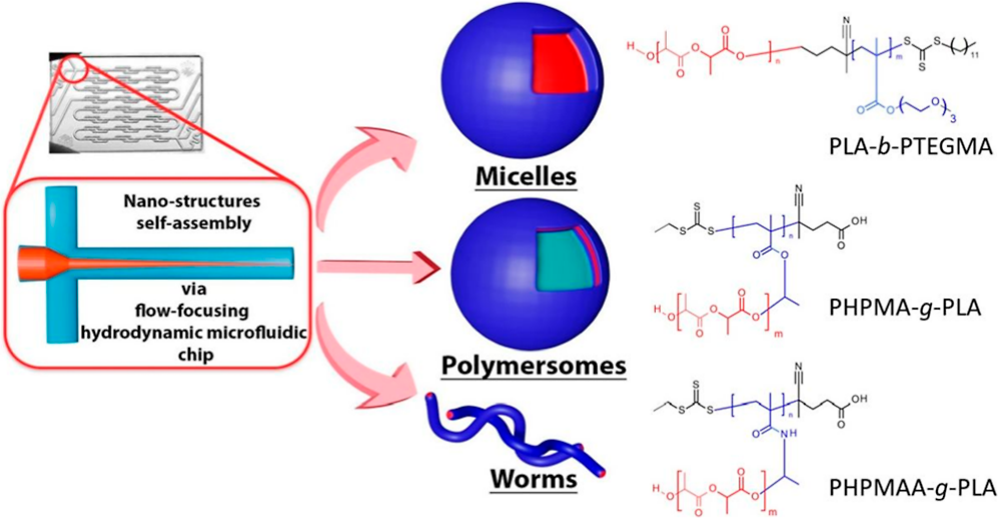

This study outlines the microfluidic (MF) controlled self-assembly of polylactide (PLA)-based linear and graft copolymers. The PLA-based copolymers (PLA-Cs) were synthesized through a convenient one-pot/one-step ROP/RAFT technique. Three distinct vinyl monomers—triethylene glycol methacrylate (TEGMA), 2-hydroxypropyl methacrylate (HPMA), and *N*-(2-hydroxypropyl) methacrylamide (HPMAA) were employed to prepare various copolymers: linear thermoresponsive polylactide-*b*-poly(triethylene glycol methacrylate) (PLA-*b*-PTEGMA), graft pseudothermoresponsive poly[*N*-(2-hydroxypropyl)] methacrylate-*g*-polylactide (PHPMA-*g*-PLA), and graft amphiphilic poly[*N*-(2-hydroxypropyl)] methacrylamide-*g*-polylactide (PHPMAA-*g*-PLA). The MF technology was utilized for the controlled self-assembly of these PLA-based BCs in a solution, resulting in a range of nanoparticle (NP) morphologies. The thermoresponsive PLA-*b*-PTEGMA diblock copolymer formed thermodynamically stable micelles (Ms) through kinetically controlled assemblies. Similarly, employing MF channels led to the self-assembly of PHPMA-*g*-PLA, yielding polymersomes (PSs) with adjustable sizes under the same solution conditions. Conversely, the PHPMAA-*g*-PLA copolymer generated worm-like particles (Ws). The analysis of resulting nano-objects involves techniques such as transmission electron microscopy, dynamic light scattering investigations (DLS), and small-angle X-ray scattering (SAXS). More specifically, the thermoresponsive behavior of PLA-*b*-PTEGMA and PHPMA-*g*-PLA nano-objects is validated through variable-temperature DLS, TEM, and SAXS methods. Furthermore, the study explored the specific interactions between the formed Ms, PSs, and/or Ws with proteins in human blood plasma, utilizing isothermal titration calorimetry.

## Introduction

**Scheme 1:**
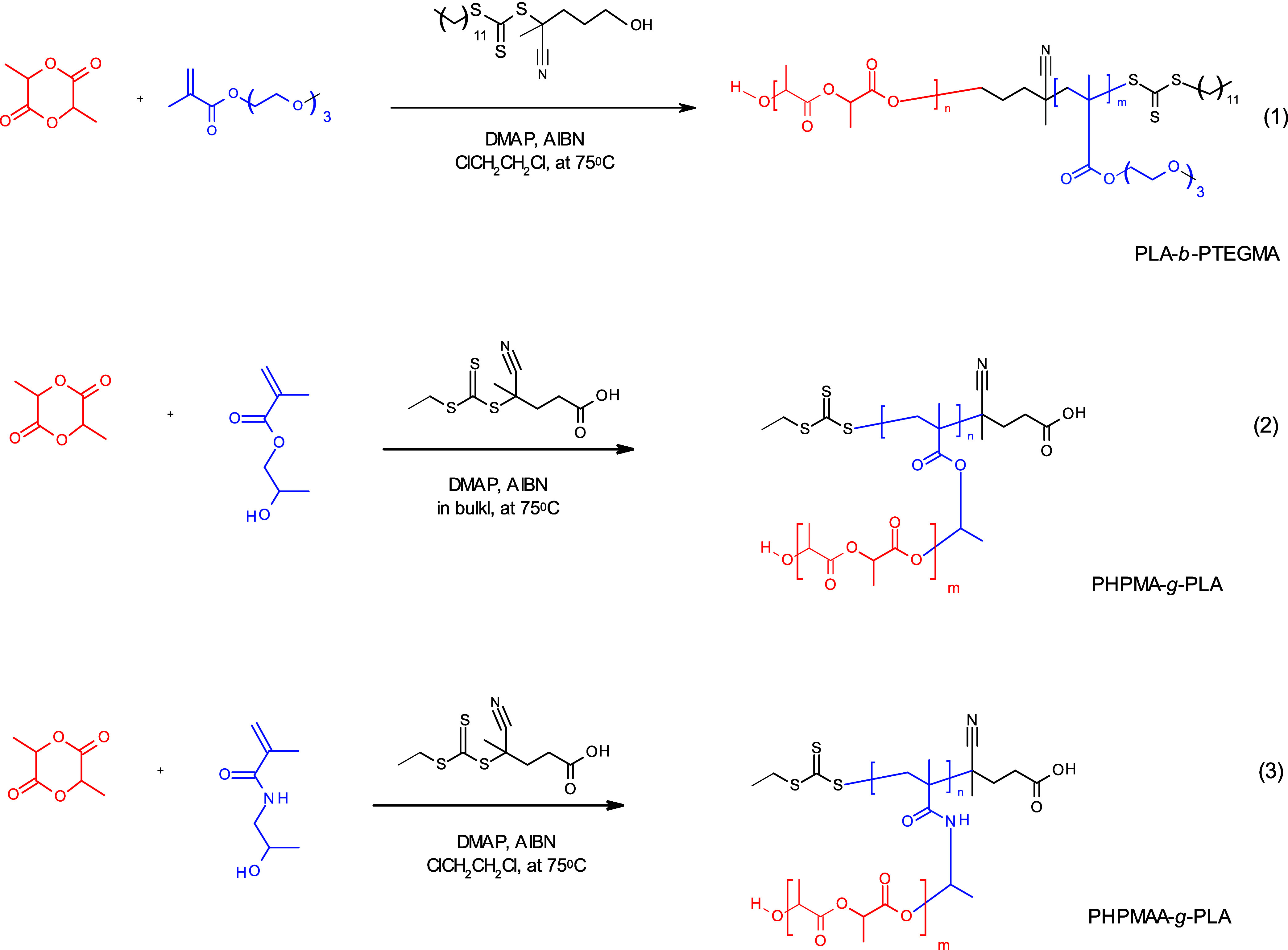
Synthetic Scheme for the Synthesis of PLA-*b*-PTEGMA Diblock Copolymer, PHPMA-*g*-PLA, and PHPMAA-*g*-PLA Graft Copolymers via One-Pot/Simultaneous

Block copolymers (BCs) have been extensively studied, both from a practical and a theoretical point of view.^1−4^ Over the last decades, most of the researcher interest have been focused on the synthesis of BCs with an amphiphilic nature and different architecture. The synthesis of well-defined BCs with a high degree of molecular, structural, and compositional homogeneity is typically achieved through the use of classic anionic and cationic polymerization methods, as well as the more recent controlled/living radical polymerization techniques. Sequential polymerization reactions are most commonly used for such syntheses.^5,6^ Of particular interest is the extremely challenging combination of two or more “living” polymerization techniques in a single step to copolymerize chemically different monomers that are otherwise noncopolymerizable.^7−9^ For example, olefin monomers and cyclic ester monomers, even within each type, are also difficult to copolymerize under identical conditions. One-pot simultaneous protocols utilize dual initiators, i.e., a reversible addition–fragmentation chain transfer (RAFT) agent bearing a hydroxyl function in either the Z- or R-position that is capable of initiating the ring-opening polymerization (ROP) of cyclic esters.^10,11^ Exploring the one-pot/simultaneous ROP/RAFT polymerization of LA with a vinyl monomer appears to be promising for the synthesis of new amphiphilic biodegradable PLA-based copolymers with different topologies.^12,13^ However, over the last decades, the synthesis of amphiphilic BCs (AmBCs) with different properties has attracted the interest of many researchers. It is well-known that their ability to undergo spontaneous self-assembly in aqueous solutions has shown great potential in a wide range of applications, such as in the pharmaceutical, medical, cosmetic, and gene delivery fields.^14−17^ Several parameters could be affected by the different morphologies of nano-objects, such as spheres, worms, polymersomes, toroids, and more complex shapes.^18,19^ For example, the chemical nature of the monomers incorporated, composition, and total molecular weight could suggest the morphology of the corresponding nanoparticles (NPs). On the other hand, specific conditions, such as temperature,^20,21^ concentration, pH,^22^ and ionic strength,^22,23^ determine the type of morphology as well. To overcome this issue, the major advantage of AmBCs prepared from a huge variety of different monomers is that they have the desired properties corresponding to their specific applications.^24^ Indeed, thermoresponsive polymers are widely used for biomedical applications, including drug delivery,^25−27^ scaffolds for tissue engineering,^28−30^ and gene delivery vehicles.^31^ The most well-known and extensively researched thermoresponsive polymer is poly-*N*-isopropylacrylamide) (PNIPAM), which has a lower critical solution temperature (LCST) in the range of 32–35 °C.^32,33^ Human body temperatures are usually in the range of 35–37 °C, which makes it the most capable trigger system. Another example of thermoresponsive polymers are oligo(ethylene glycol) methacrylates, which allow precision tailoring of LCST dependent upon pendant chain length and end group and are believed to be biocompatible and suitable for drug delivery applications in a manner analogous to linear PEG,^34−42^ 2-hydroxypropyl methacrylate (HPMA), and so on. Interestingly, PHPMA-based diblock copolymer NPs do not have a typical LCST profile, but they are weakly thermosensitive, which leads to shape-shifting behavior when adjusting the solution temperature.^43^ Armes et al. conducted extensive studies on an aqueous dispersion of poly([*N*-(2-hydroxypropyl) methacrylate (PHPMA)]-based diblock copolymers. These studies have demonstrated that the copolymers exhibit spheres, worms, or vesicles morphology simply by adjusting the solution temperature.^38,44,45^ It should be noted here that the thermoresponsive behavior of PHPMA strongly depends on its degree of polymerization (DP). At relatively high DP, PHPMA is no longer thermoresponsive.^37,44,46^ In contrast, the study selected poly(*N*-(2-hydroxypropyl)methacrylamide) (PHPMAA) as a representative of nonthermosensitive polymers. PHPMAA stands out for its highly hydrophilic, nontoxic, and biocompatible properties. Additionally, it possesses protein-repelling characteristics and offers a prolonged blood circulation lifetime.^47,48^ In 2010, Zentel et al. proposed a new route toward the synthesis of functional PLA-*b*-PHPMAA via the combination of ROP of LA and conversion into a chain transfer agent for the subsequent RAFT polymerization.^49^ A one-step/simultaneous RAFT/ROP approach was found for the first time by our team for the copolymerization of PLA with different vinyl monomers.^50−52^ It is well-known that PLA is a hydrophobic, aliphatic, biodegradable, and biocompatible synthetic biomaterial.^53−56^ It has also been utilized in biomedical applications including sutures, bone screws, and tissue engineering scaffolds.^57,58^ Nevertheless, controlling the size, shape, and polydispersity of soft-matter self-assembled nanostructures is an important strategy, which is why the application of microfluidic (MF) devices is greatly necessary.^59^ Indeed, MF methods featuring precise fluid manipulation have quickly become powerful and versatile for manufacturing NPs in a highly controlled manner, which is of great importance for nanoencapsulation, biomimics, and microreactors.^60^ Considering the aforementioned factors, we were motivated to develop a novel metal-free one-pot/one-step approach. Here, we combined RAFT/ROP techniques to create biodegradable and biocompatible PLA-based BCs. For this purpose, three distinct vinyl monomers: triethylene glycol methacrylate (TEGMA), HPMA, and HPMAA were used. These versatile components were instrumental in the creation of a diverse range of polymers, including the thermoresponsive linear polylactide-*b*-poly(triethylene glycol methacrylate) (PLA-*b*-PTEGMA), the pseudothermoresponsive poly([*N*-(2-hydroxypropyl) methacrylate-*g*-polylactide) (PHPMA-*g*-PLA), and the intriguing amphiphilic poly([*N*-(2-hydroxypropyl) methacrylamide-*g*-polylactide) (PHPMAA-*g*-PLA) copolymers (see, Scheme 1). Our study aims to explore the self-assembling behavior of three AmBCs using a MF chip system. Additionally, we aim to conduct a pioneering comparison of the interplay between polymer chemistry, thermoresponsivity, and the architecture formed through self-assembly on a MF chip. Three PLA-based BCs were obtained with similar molecular weights but varying composition, structural properties, and topology. To the best of our knowledge, this is the first report demonstrating precise control of kinetic processing by adjusting the final polymer concentration (*C*_final_), leading to the self-assembly of these three AmBCPs into micelle-like spheres (Ms), Ps, and worms (Ws). Furthermore, the specific interactions between the obtained NPs and proteins in human blood plasma were studied by isothermal titration calorimetry (ITC). We are confident that unraveling these rules and relationships governing structure, thermoresponsivity, and architecture formation will open up new avenues for customizing the self-assembly of nanosystems for biomedical applications.

## Materials and Methods

### Materials

The monomer 3,6-dimethyl-1,4-dioxane-2,5-dione (d,l-lactide, LA, 99%) underwent several recrystallization steps from ethyl acetate before use. HPMA and methacryloyl chloride were purified via vacuum distillation prior to usage. The monomer *N*-(2-hydroxypropyl)methacrylamide (HPMAA) (as depicted in Figure S1) was synthesized following the procedure outlined in ref (61) The RAFT agent 4-cyano-4-(((ethylthio)carbonothioyl)thio)pentanoic acid (CEPA) (as shown in Scheme S1 in the Supporting Information) was synthesized in accordance with literature in ref (62). Detailed synthesis procedures for all compounds are provided in the Supporting Information file. Triethylene glycol methyl ether methacrylate (TEGMA, 95%) was purified using an inhibitor remover (Aldrich #311332) with stirring for 5 min, followed by filtration to remove the inhibitor remover. The initiator 2,2′-azobis(2-methylpropionitrile) (AIBN, ≥98%) was purified through recrystallization from methanol. The chain-transfer agent, 4-cyano-4-[(dodecylsulfanylthiocarbonyl)sulfanyl]pentanol (CDSP), was used as received. Anhydrous 1,2-dichloroethane (Cl_2_CH_2_CH_2_Cl_2_, 99.8% purity) was distilled under an Ar atmosphere. 1,4-Dioxane of the highest commercially available purity, along with all reagents and solvents, was purchased from Sigma-Aldrich. The chemicals amino-2-propanol and 4-(dimethylamino)-pyridine (DMAP, 98%) were purchased from Fluka (Czech Republic). Regenerated cellulose dialysis membranes with a molecular-weight cutoff (MWCO) of 2 kDa were purchased from Spectra/Por.

### Characterization Techniques

A comprehensive explanation of the employed characterization techniques is provided in detail within the Supporting Information file.

### Synthesis of PLA-Based BCs with Diverse Architectures

The PLA-*b*-PTEGMA diblock copolymer,^50^ PHPMA-*g*-PLA graft copolymer (GCP),^52^ and PHPMAA-*g*-PLA GCP^51,52^ were synthesized via a one-pot/one-step protocol following previous procedures, which are detailed in the Supporting Information.

### Self-Assembly of PLA-Based BCs in Microfluidic Chips and Characterization of NPs

Nano-objects with different morphologies were produced by a MF device (Dolomite Royston, United Kingdom) and using a glass micromixer chip with 12 mixing stage microchannels of 50 μm × 125 μm (depth × width). For the self-assembly of the BCs in MF chips, Nemesys pumps (CETONI, Germany) were used (see Figure S1, in Supporting Information). The self-assembly process of the BCs PLA_35_-*b*-PTEGMA_64_ and PHPMA_75_-*g*-PLA_35_ was conducted at an initial solution concentration (*C*_initial_) of 2.0 mg L^–1^ and PHPMAA_82_-*g*-PLA_35_ at a *C*_initial_ of 5.0 mg L^–1^. The used concentration was chosen to prevent the formation of macroscopic aggregates within the MF chip. The PLA_35_-*b*-PTEGMA_64_ and PHPMA_75_-*g*-PLA_35_ copolymers were dissolved in THF, while PHPMAA_82_-*g*-PLA_35_ was dissolved in THF/MeOH (80/20) (v/v). The polymer solutions were pumped through the middle channel, and miliQ water was pumped through the side channels using two separate liquid steams controlled via computer software. For all cases, the final concentration (*C*_final_) should be achieved at 20.0 mg mL^–1^. The flow rates were adjustable parameters, and the polymer colloids were obtained after evaporation of the organic solvent by a rotary vacuum evaporator. The supramolecular polymer assemblies were characterized by dynamic light scattering (DLS), transmission electron microscopy (TEM), and small-angle X-ray scattering (SAXS) techniques, as described in detail.

## Results and Discussion

### Synthesis of Polyester PLA-Based Copolymers and Their Self-Assembly in MF Chips

Herein, we showcase kinetically controlled self-assembly in MF channels. We use various PLA-based copolymers predissolved in organic solvents, achieving a *C*_final_ of 20.0 mg mL^–1^ for all. The size, shape, and polydispersity of the self-assembled nanostructures were carefully controlled using the MF-assisted method.^47^ To achieve precise control over these parameters, it was imperative to fine-tune kinetic factors during the self-assembly process.

In our recent published papers,^50−52^ we showcased the synthesis of a novel category of BCPs with linear and nonlinear architecture. Likewise, all were synthesized via a metal-free one-pot/simultaneous ROP and RAFT approach. The BCPs consist of a constant block of hydrolyzable aliphatic biodegradable polyester (PLA), and a second block of methacrylic and/or methacrylamide, such as PTEGMA, PHPMA, and PHPMAA. The variation involved maintaining a similar ratio of the blocks while ensuring that they have nearly identical molecular weights. Using this set of diverse building blocks, various copolymers were prepared for all classes of amphiphiles outlined in Scheme 1. The BCPs are as follows: thermoresponsive polylactide-*b*-poly(triethylene glycol methacrylate) (PLA-*b*-PTEGMA) diblock copolymer, pseudothermoresponsive poly([*N*-(2-hydroxypropyl)]methacrylate*-g*-polylactide (PHPMA-*g*-PLA), and poly([*N*-(2-hydroxypropyl)]methacrylamide-*g*-polylactide (PHPMAA-*g*-PLA) GCPs.

**Figure 1:**
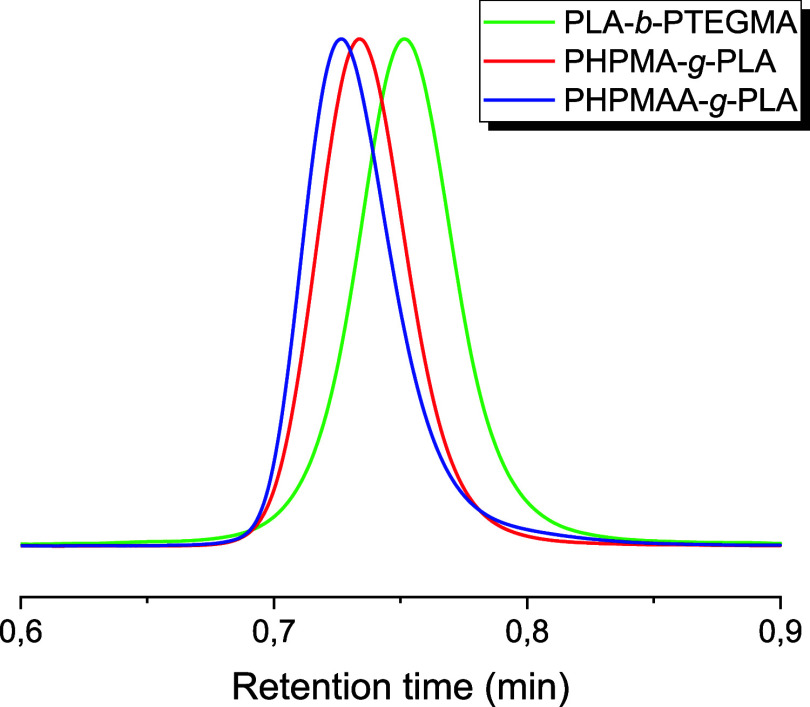
SEC chromatograms in DMF of the PLA-*b*-PTEGMA diblock copolymer (green curve), PHPMA-*g*-PLA (red curve), and PHPAA-*g*-PLA graft copolymers (blue curve).

The SEC chromatograms of all investigated PLA-based copolymers clearly show that the obtained curves are monomodal, symmetric, and exhibit no competitive side reactions. Notably, the molecular-weight distributions (*D̵*) were relatively narrow: polydispersities ranged between 1.13 and 1.21 (Table 1), which indicates that the combination of ROP and RAFT polymerization in a one-pot/one-step protocol proceeded as a living process, and the obtained PLA-based BCPs have controlled structures (see Figure 1).

**Table 1:** Molecular Weight Data of All BCPs Synthesized via Metal-Free One-Pot/One-Step ROP/RAFT Polymerization

BCPs	[LA]_0_/[M_RAFT_]_0_ feed ratio	*M*_n_, SEC (g·mol^–^^1^)^a^	*D̵*
PLA-*b*-PTEGMA	35/64	14,300	1.21
PHPMA-*g*-PLA	35/75	20,100	1.20
PHPMAA-*g*-PLA	35/85	21,900	1.13

a Determined by SEC in DMF as the eluent poly(methyl methacrylate) (PMMA) standards.

### Formation of PLA-*B*-PTEGMA Ms in MF Channels

**Figure 2:**
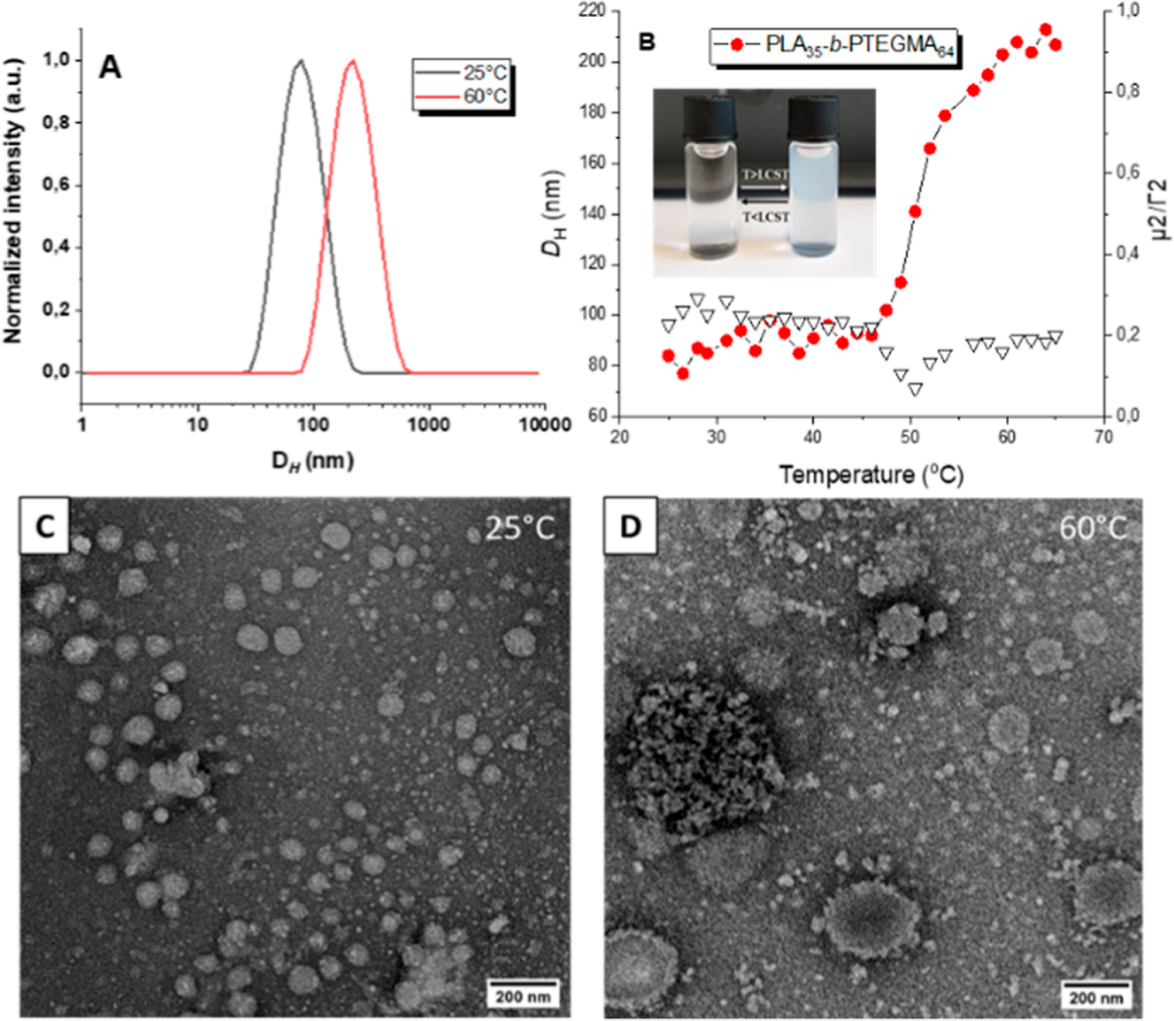
(A) DLS plot for the hydrodynamic diameters of PLA-*b*-PTEGMA Ms at 25 and 60 °C; (B) temperature dependence of hydrodynamic radius (*D*_H_) of diblock polymer in aqueous solution; and TEM images of self-assembled PLA-*b*-PTEGMA at (C) 25 °C and (D) 60 °C.

Herein, the linear thermoresponsive PLA-*b*-PTEGMA diblock copolymer (T-BCP) assembled into thermodynamically stable micelle-like (Ms) spherical nano-objects at a flow rate of 300/100 μL min^–1^ from the WP to OP, achieving a *C*_final_ of 20.0 mg mL^–1^. It is well-known that T-BCPs sensitive to temperature changes in the environment are characterized by a LCST-type phase transition. This is a well-established feature for thermoresponsive polymers and polymeric nano-objects that display an LCST-type phase transition.^11,16,63,64^Figure 2A shows the particle size distribution of the copolymer at room temperature and 60 °C. According to DLS analysis, at lower temperatures (*T* < LCST), the hydrodynamic diameter (*D*_H_) values of the thermoresponsive micelles are approximately 78 nm, with a particle size distribution (PDI = 0.23), see Table 2. At higher temperatures, reaching 60 °C (*T* > LCST), the hydrodynamic diameter (*D*_H_) values rapidly increase to 213 nm (PDI = 0.126), see Table 2. The DLS analysis showcased the lactate moieties’ capacity to modulate the cloud point, which is governed by the thermoresponsive block (PTEGMA). Additionally, prior research conducted by our team revealed that the length of the hydrophobic PLA block influenced cloud point concerning the length of the PTEGMA block.^50^

**Table 2:** Physico-chemical Characteristics of the PLA-*b*-PTEGMA, PHPMA-*g*-PLA, and PHPMAA-*g*-PLA NPs Produced by Microfluidics

N	copolymers	flow rate (OP/WP)	*D*_H_/nm, (PDI)^a^		morph^b^	*D*/nm^c^
1	PLA-*b*-PTEGMA thermoresponsive block copolymer	THF/H_2_O 100/300	25°C	78 (0.23)	Ms	70–80
			60°C	213 (0.126)	A	200–300
2	PHPMA-*g*-PLA pseudothermoresponsive graft copolymer	THF/H_2_O 100/300	5°C	124 (0.06)	SMs	70–80
			25°C	152 (0.03)	PSs	100–150
			60°C	175 (0.04)	PSs-A	150–200
3	PHPMAA-*g*-PLA amphiphilic graft copolymer	THF/MeOH/H_2_O (80/20 v/v) 100/300		140 (0.34)	Ws	15 ± 3.2

a Hydrodynamic diameter and dispersity from DLS.

b Nanoparticle morphology from TEM (Ms = micelles, A = aggregates, PSs = polymersomes, SMs = spherical micelles, and Ws = worms).

c Particle diameter from TEM.

The cloud point and phase separation behavior of PLA-*b*-PTEGMA Ms was subsequently explored by variable-temperature DLS analysis. The temperatures ranged from 25 to 65 °C in 1.5 °C intervals (refer to Figure 2B), depicting the dependence of the Z-average *D*_H_ on temperature. For Ms formulation, heating above a cloud point significantly increased the *D*_H_. This was accompanied by the formation of larger aggregates due to decreased particle solubility above their LCST. The phase transition temperature is reported here as the *T*_CFT_, recorded as the *D*_H_ value at the onset of exponential particle size increase. The specified cloud point value of the PLA-*b*-PTEGMA exemplified in this study is around 50 °C, and *T*_CFT_ is about 55 °C; see Figure 2B. Imaging of T-BCP self-assembled by TEM confirmed the presence of uniform spherical particles-Ms. In order to further additionally improve the temperature-responsive nature of the PLA-*b*-PTEGMA Ms explored by variable-temperature TEM.

Indeed, Figure 2C shows the TEM image of PLA-*b*-PTEGMA Ms at 25 °C (*T* < LCST), where the well-defined spherical NPs can be observed. As expected, when the temperature increased to 60 °C (*T* > LCST), the Ms tended to form aggregates of large size, as shown in Figure 2D, which can be attributed to the transition of PTEGMA chains from hydrophilicity to hydrophobicity. Overall, the DLS results are in good agreement with the TEM images and provide further evidence of the morphology of the resulting NPs.

**Figure 3:**
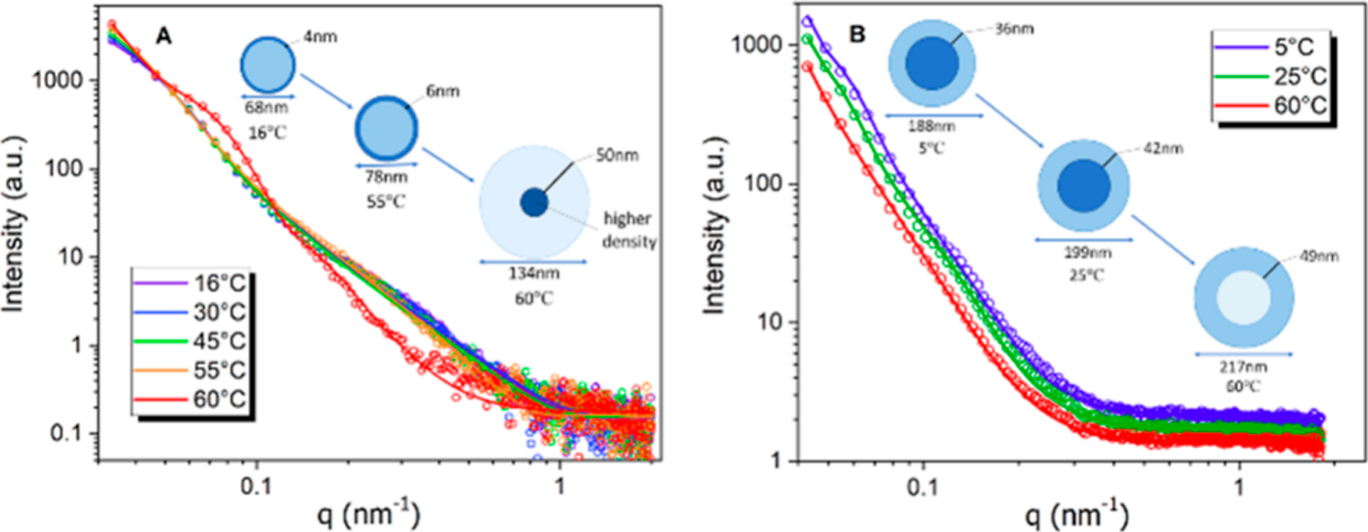
Selected SAXS data (colored circles) recorded for nano-objects prepared by MF of (A) PLA-*b*-PTEGMA on heating from 16 to 60 °C and (B) PHPMA-*g*-PLA at temperatures of 5, 25, and 60 °C.

Furthermore, to demonstrate the thermoresponsivity of PLA-*b*-PTEGMA diblock copolymer, the powerful analytical technique SAXS was used. The thermally induced micelle-to-micelle aggregation transition was verified by a series of experiments that yielded five characteristic SAXS patterns at different temperatures: 16, 30, 45, 55, and 60 °C, respectively. The experimental SAXS patterns obtained for the original T-BCP Ms are shown in Figure 3A.

These patterns were fitted using the core–shell model,^65^ and the obtained parameters at room temperature indeed demonstrated that PLA-*b*-PTEGMA T-BCP NPs have a micellar structure with 63 nm in diameter and a thin shell of about 3.8 nm in thickness with a slightly lower scattering length density compared to the core. With increasing temperature, the size of the Ms increased slightly to about 66.8 nm and a thin shell of about 6.3 nm in thickness at 55 °C. When the temperature was increased even further (to 60 °C), a significant shift in the morphology of the particles was observed—the size of the whole particle increased two-fold, while the core shrunk to about half its size and noticeably increased its scattering length density (see Table 3). Even after aggregation, the particles retained a predominantly spherical shape. However, their polydispersity notably increased (refer to Figure 2D), thus warranting the application of a core–shell model with a log–normal polydispersity function for fitting.

**Table 3:** Parameters of the Core–Shell Model for PLA-*b*-PTEGMA and PHPMAA-*g*-PLA NPs at Various Temperatures with a Log–Normal Distribution of Core and Shell Radii to Account for the Polydispersity of the Samples

PLA-*b*-PTEGMA						PHPMAA-*g*-PLA		
*T* [°C]	16	30	45	55	60	5	25	60
*r*_core_ [nm]	30.1	31.5	32.0	33.4	17.0	58.1	57.7	59.2
*r*_shell_ [nm]	4.4	3.8	4.6	6.3	50.4	35.7	41.7	49.3
ρ_core_/ρ_solvent_	1.23	1.24	1.26	1.29	2.25	1.66	1.65	1.18
ρ_shell_/ρ_solvent_	1.71	1.80	1.70	1.66	0.92	1.56	1.55	1.48

### Formation of PSs in MF Channels

Through the use of the MF method, the pseudothermoresponsive PHPMA-*g*-PLA GCP self-assembles to produce PSs with a controlled size. The flow rate was held constant (100 μL min^–1^) while the flow rate of the aqueous phase under flow velocity ratio was 300 μL min^–1^, resulting in vesicular shape with particle size shown in Table 2. THF was used as an organic solvent to solubilize the copolymer.

**Figure 4:**
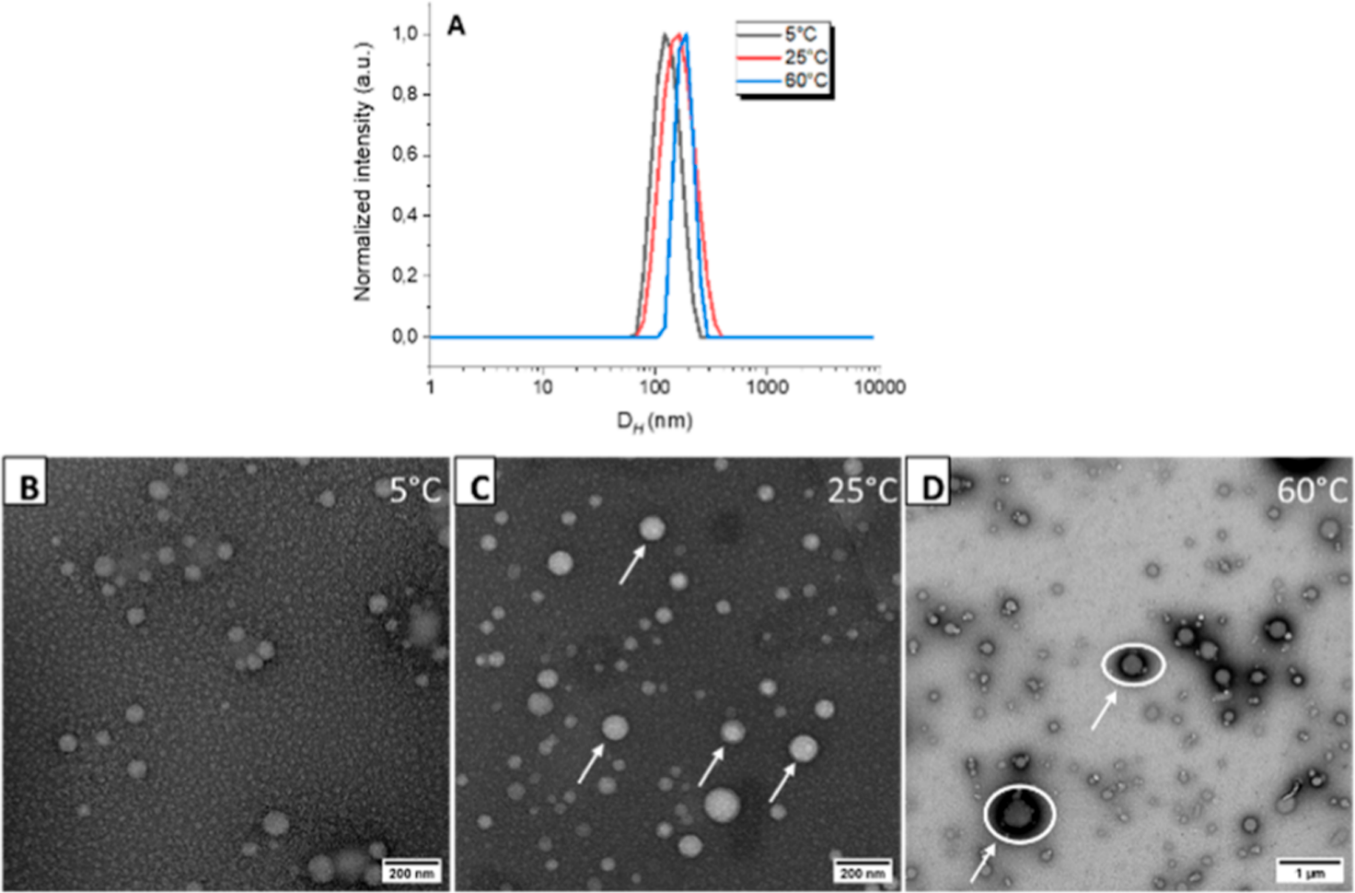
(A) The plots depict the hydrodynamic diameter of the NPs at various temperatures, alongside TEM micrographs of self-assembled PHPMA-*g*-PLA at (B) 5 °C, (C) 25 °C, and (D) 60 °C.

It is well-known that nano-objects containing PHPMA blocks exhibit nontypical thermoresponsive behavior, i.e., (they do not possess LCST)^14,31,66^ so-called pseudothermoresponsive polymers. Inspired by the extensive research carried out by Armes et al. on PHPMA-based NPs, we invented a new shell-forming PHPMA block containing a core PLA-grafted block. In relation to the above, we conducted proof-of-concept experiments to demonstrate the thermosensitivity of the graft PHPMA-*g*-PLA NPs. The thermoresponsive properties of the PSs were then checked by monitoring the hydrodynamic diameter with DLS. Three different temperatures 5, 25, and 60 °C were selected. As shown in Figure 3A, the DLS intensity profile of the obtained PSs at 25 °C shows just one distinct population (*D*_H_ = 152 nm) with a relatively narrow particle size distribution (PDI = 0.03; Figure 4A, Table 2).

The mean hydrodynamic diameter was slightly changed with increasing the temperature up to 60 °C (*D*_H_ = 175 nm, PDI = 0.04); see Table 2. It is indicating that the PHPMA-*g*-PLA PSs did not exhibit thermoresponsive behavior at high temperatures. When the temperature was decreased to 5 °C, only a very modest decrease in the *Z*-average diameter (*D*_H_ = 124 nm, PDI = 0.06) was found. A likely reason for this change is due to the fact that the PHPMA polymer becomes significantly more hydrated when the solution temperature decreases, as previously shown by Armes et al.^18,45^ Despite this minor issue, it still provides an effective strategy to tune the GCP vesicular morphologies and structures. These observations suggest that the micellar morphology of the study NPs at low temperatures could undergo changes. Following this procedure, the PHPMA-*g*-PLA aqueous dispersion was performed at temperatures of either 5 °C (utilizing a refrigerator), 25 °C (at room temperature), or 60 °C (with the assistance of an oven), after a 24 h equilibration period. The homogeneity of the sample was subsequently confirmed through TEM micrographs (Figure 4B–D; see Table 2). The TEM study validated our hypothesis regarding a morphological change. Theoretically, within the membrane-forming PHPMA block, an increase in environmental temperature from 25 to 60 °C is anticipated to induce a transition to a hydrophobic state. This transition leads to a reduction in the packing parameter *P*, which in turn triggers morphological changes. As a result, this phenomenon explains the formation of vesicle-to-vesicle aggregates that have been observed.^37,67^ Unlike our scenario, where no noticeable alteration in the micellar-vesicle morphology was observed upon heating to 60 °C, there was merely an increase in the *D*_H_ by approximately 20 nm (Figure 4D). The larger size of the molecules measured using DLS at 60 °C could be attributed to their partial aggregation, which we can observe in TEM (Figure 4D).

For example, at rapid cooling from 25 to 5 °C, there was a partial change in the morphology from the mixture of vesicles and micelles to pure micelles with a negligible change in the particle size (Figure 4B,C). On the basis of the structural transformation of spherical micelles (SMs) along with temperature adjustments, it is hypothetically possible to conclude that SMs are connected by PHPMA domains. The NPs studied by DLS at different temperatures and the change in *D*_H_ are well-consistent with TEM results (Figure 4). Regrettably, this did not facilitate the recognition of the PHPMA-*g*-PLA GCP under investigation as a suitable candidate for generating nanoformulations displaying spherical, worm-like, vesicular, or lamellar morphology within the specified temperature range (5, 25, and 60 °C).

To confirm the PS or SM morphologies assigned by TEM analysis, SAXS patterns were recorded for PHPMA-*g*-PLA aqueous copolymer dispersion at 5, 25, and 60 °C (see Figure 3B). Fitting this SAXS pattern to the spherical micelle model^65^ indicated an overall sphere diameter (*D*_sphere_). The characteristics of PHPMA-*g*-PLA suggest that the particles exhibit the characteristics of larger vesicles (approximately 200 nm in diameter at room temperature) with a more substantial shell (40 nm) possessing a greater scattering length density in comparison to the core. As the temperature increases, the shell thickness also increases, resulting in a growth in the overall size of the vesicle. When the temperature was raised to 60 °C, the scattering length density of the core decreased to a level similar to that of water. This observation could be interpreted as an indication of water entering the interior of the vesicle (see Table 3).

### Formation of Ws in MF Channels

**Figure 5:**
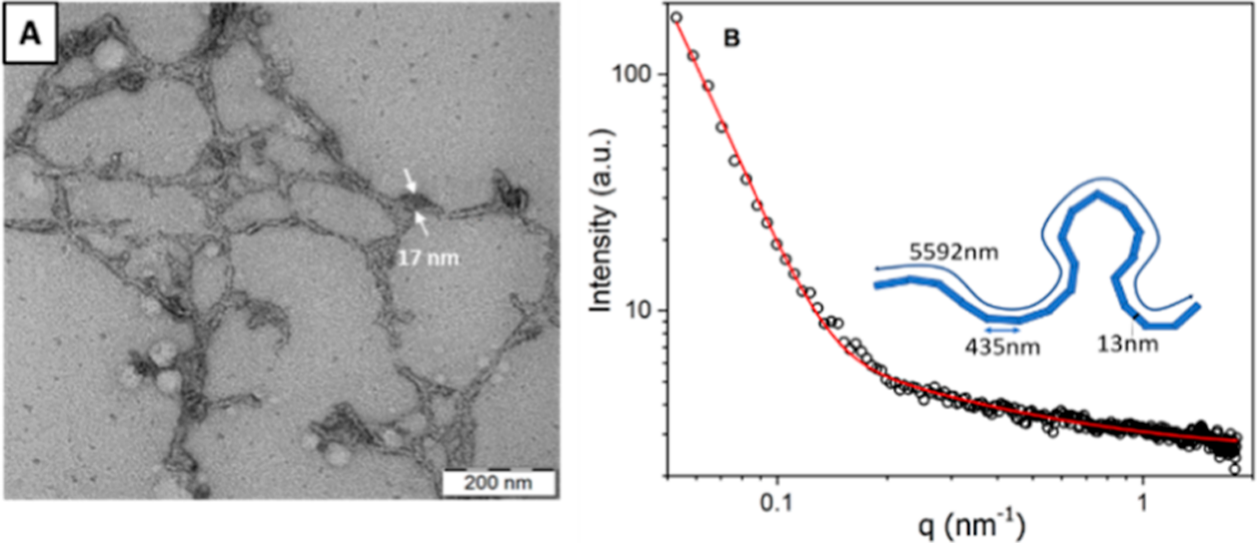
(A) TEM image and (B) SAXS data of recorded for the self-assembled of PHPMAA-*g*-PLA Ws. Adapted with permission under a Creative Commons [CC-BY 3.0] from ref (51) Copyright 2024 Royal Society of Chemistry.

In our recent publication,^51^ we highlighted the significant impact of self-assembly within the MF technique, particularly evident with a typical amphiphilic PHPMAA-*g*-PLA GCP. This copolymer enabled the formation of nano-objects with a unique worm-like (Ws) morphology at a concentration of *C*_final_ 20.0 mg mL^–1^. Achieving such a morphology is rare, especially with amphiphilic BCs rooted in PLA and PHPMAA. Typically, NPs derived from these copolymers exhibit spherical shapes. Our recent breakthrough, made possible by the MF technology, demonstrates the successful generation of nano-objects with a worm-like morphology using amphiphilic PLA-*g*-PHPMAA GCPs. The production of these worm-like structures was accomplished using a mixture of THF/MeOH (80/20) (v/v) as the organic solvent to dissolve the GCP. The solution was pumped at a flow rate of 100 μL min^–1^, and the flow diffusion occurred under a flow velocity ratio of the aqueous phase (300 μL min^–1^). Remarkably, the MF technique enabled the creation of uniform NPs exhibiting the distinctive worm-like morphology, a feature not typically associated with this method. In fact, there are very few instances in the literature where the formation of worm-like micelles has been successfully achieved using the MF approach.^60,68^ Furthermore, it is worth highlighting that no instances of worm self-assembly using PLA–PHPMAA copolymers have been reported, even through conventional NP preparation methods like emulsion, dialysis, and others. The assembly of worm particles from PHPMAA-*g*-PLA copolymers within MF channels is evident in both the DLS profile (not shown) and the TEM micrograph (Figure 5A). The DLS intensity profile displayed a monomodal size distribution with an apparently *D*_H_ = 140 nm and a relatively broad polydispersity (PDI = 0.34). This is a characteristic feature often observed in nano-objects exhibiting worm-like morphology.^68−71^Figure 5A depicts well-defined worms nano-objects, and it is estimated from the TEM image to be of the order of about 15 nm. These PHPMAA-*g*-PLA worms were further characterized by SAXS. For an optimal fit to the SAXS pattern, a combination of the WormLikeChainEXV model described by Pedersen^72^ (to characterize the worm-like chains) and the extended Guinier law model^73^ (to describe the corona block) was used. The effect of solvent (water) was approximated by the constant function *I*_H_2_O_ = 3.41. The mean worm contour length or total length (*L*_w_) was determined to be 5592 nm (see Figure 5B).

The mean worm width (*W*_w_) was calculated to be 13 nm, according to the circular worm cross-section. The resulting value corresponds to that estimated from TEM images (for which *W*_w_ = 15 ± 3.2 nm). For this purpose, the equation *W*_w_ = 2*R*_sw_ + 4*R*_g_ was applied, where *R*_sw_ represents the radius of the worm core cross section and *R*_g_ represents the radius of gyration of the corona chains (PHPMAA). Based on the SAXS pattern fit, the Kuhn length–length of two neighboring rigid sections *RL*_w_ is about 870 nm (one segment = 435 nm), and the *R*_g_ of the corona block was determined to be 1736 nm (*d* = 3472 nm).

**Figure 6:**
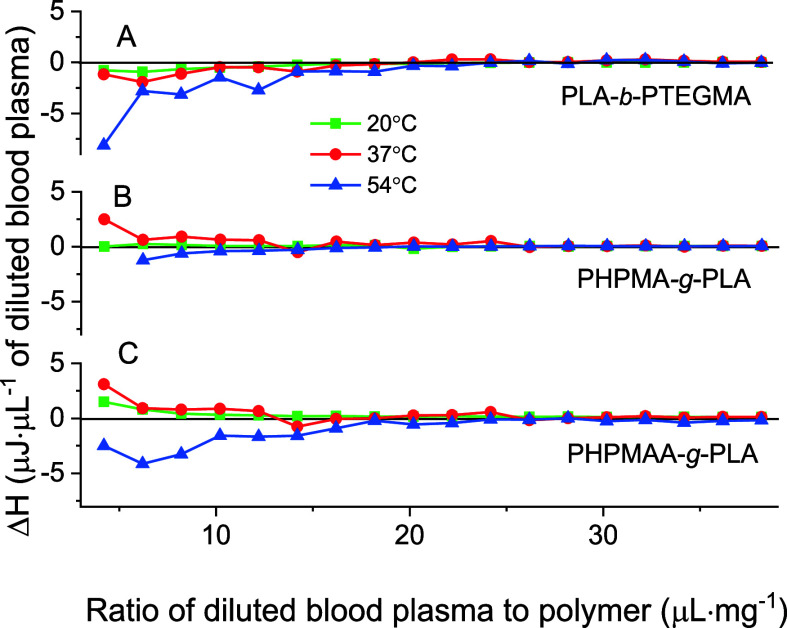
ITC of polymers (A) PLA-*b*-PTEGMA, (B) PHPMA-*g*-PLA, and (C) PHPMAA-*g*-PLA NPs in ultrapure water with human blood plasma, diluted to 10% vol/vol with PBS at 20, 37, and 54 °C.

The abundant proteins found in blood plasma were used to investigate the interaction of the obtained NPs within MF. ITC experiments were conducted to assess the binding affinity of these proteins to PLA-*b*-PTEGMA, PHPMA-*g*-PLA, and PHPMAA-*g*-PLA nano-objects. The ITC experiments with diluted blood plasma were carried out at three different temperatures: the ambient temperature of 20 °C, the physiological temperature of 37, and 54 °C. At both 20 and 37 °C, all three polymers exhibited minimal interaction with the proteins in blood plasma, as depicted in Figure 6. A slight initial endothermic signal observed during the titration process, particularly for PHPMA-*g*-PLA and PHPMAA-*g*-PLA GCPs, indicates the dehydration of polar groups present either in the polymers or in the proteins. However, at 54 °C, an exothermic signal was observed for all three polymers. This signal can be tentatively attributed to the binding of certain proteins through hydrophobic interactions.

It is noteworthy that the hydrophobicity of the polymers follows the order PLA-*b*-PTEGMA > PHPMAA-*g*-PLA > PHPMA-*g*-PLA. Nonetheless, the measured thermal effects resulting from the titration of polymeric solutions with diluted human blood plasma were relatively small, making it challenging to definitively confirm the binding of proteins across all the examined temperatures. As a result, it can be concluded that the NPs do not readily interact with the proteins present in human blood, thereby exhibiting nonfouling behavior.

## Conclusions

In summary, the combined utilization of ROP/RAFT “living” polymerization techniques within a one-pot/simultaneous protocol presents a highly versatile synthetic strategy for producing biocompatible linear and grafted amphiphilic block copolymers. This demonstrates that the cyclic ester LA and the three distinct methacrylic monomers, namely, TEGMA, HPMA, and *N*-(2-hydroxypropyl) methacrylamide (HPMAA), serve as suitable comonomers that can effectively participate in ROP/RAFT processes. The central focus of this research lies in the kinetic-controlled self-assembly of three block copolymers: PLA-*b*-PTEGMA, PHPMA-*g*-PLA, and PHPMAA-*g*-PLA, using a capillary-based MF chip. Within the MF framework, the morphology and size of the self-assembled nanopharmaceuticals are determined by the nature and composition of the corresponding copolymers.

Among the entire spectrum of copolymers assemblies, particle size can be conveniently adjusted by modifying either the total flow velocity of water and the organic solvent, or their flow velocity ratio. Depending on the specific AmCPs and the self-assembly protocol, various morphologies can be achieved using in all cases the same *C*_final_. Specifically, PLA-*b*-PTEGMA and T-BCP self-assemble into monodisperse (PDI <0.20) micelles with the desired size (*D*_H_ = 78 nm) at room temperature. Upon raising the temperature, the particle size increases (*D*_H_ = 213 nm, PDI = 0.126), leading to the aggregation of micelles. The observed temperature range for this aggregation phenomenon spans from 50 to 60 °C.

Variable-temperature DLS and TEM investigations validate that the pseudothermoresponsive PHPMA-*g*-PLA experiences increased solvation and/or mobility at lower temperatures. TEM analysis reveals the presence of PSs at 25 °C, undergoing an order–order transition to spherical micelles upon cooling to 5 °C. Subsequent heating to 60 °C triggers the aggregation of the PSs.

This study presents one of the initial instances of PHPMAA-*g*-PLA nanoformulations exhibiting a distinct worm morphology, characterized by their stable self-assembled structures.

With their inherent biocompatibility, these novel nanomaterials hold promise as platforms for crafting unique nanovectors.

## Supporting Information Available

Details of synthesis and characterization techniques (PDF)
