# Supplementary material for: Microfluidic Controlled Self-Assembly of Polylactide (PLA)-Based Linear and Graft Copolymers into Nanoparticles with Diverse Morphologies
Source: ACS Polym Au. 2024 May 31;4(4):331–41. doi: 10.1021/acspolymersau.4c00033 (PMC11328328; doi:10.1021/acspolymersau.4c00033)
Supplement: Supplementary file 1 — lg4c00033_si_001.pdf [file lg4c00033_si_001.pdf]

Electronic Supplementary Material

Microfluidic Controlled Self-Assembly of Polylactide (PLA)-Based Linear and Graft Copolymers into Nanoparticles with Diverse Morphologies

Svetlana Lukáš Petrova, Vladimir Sincari, Ewa Pavlova, Václav Pokorný, Volodymyr Lobaz and Martin Hrubý*

Institute of Macromolecular Chemistry v.v.i., Academy of Sciences of the Czech Republic, Heyrovsky Sq. 2, 162 06 Prague 6, Czech Republic

Synthesis of monomer (HPMA) and CTA (CTA-COOH)

1) *N*-(2-hydroxypropyl) methacrylamide (HPMA)

Na_2CO_3 (12.61 g, 119 mmol) was suspended in DCM (28 mL). The mixture was cooled to -10 ^oC and amino-2-propanol (8.35 mL, 108 mmol) was added. Freshly distilled methacryloyl chloride (10.56 mL, 108 mmol) in 11 mL of DCM was added dropwise with the solution kept between 25 and -10 ^oC (ethanol/dry ice mixture) and vigorously stirred. The mixture was stirred for 20 min at -5 ^oC and then left stirring to equilibrate to room temperature. Following filtration, the filtrate was dried twice with anhydrous Na_2SO_4 and concentrated until formation of crystallization seeds. The product was left to crystallize at -20 ^oC overnight, washed with cold DCM and recrystallized from acetone The crystals were dried in vacuo (16.1 g, 85%).

^{1}H-NMR (600 MHz, DMSO, δ, ppm): 1.00 (d, 3H, CH(OH)-C*H*$_3$), 1.85 (q, 3H, C(CH$_2$)C*H*$_3$)), 3.05 (m, 2H, NH-C*H*$_2$), 3.68 (m, 1H, C*H* (OH)), 4.68 (d, 1H, CH (*OH*)), 5.31 (m, 1H, =C*H*$_2$), 5.65 (m, 1H, =C*H*$_2$), 7.80 (s, 1H, N*H*-CH$_2$) (see Figure S1 for assignments).

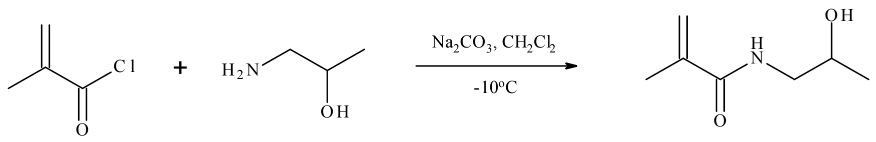

Scheme S1. Synthesis of the *N*-(2-hydroxypropyl) methacrylamide (HPMA).

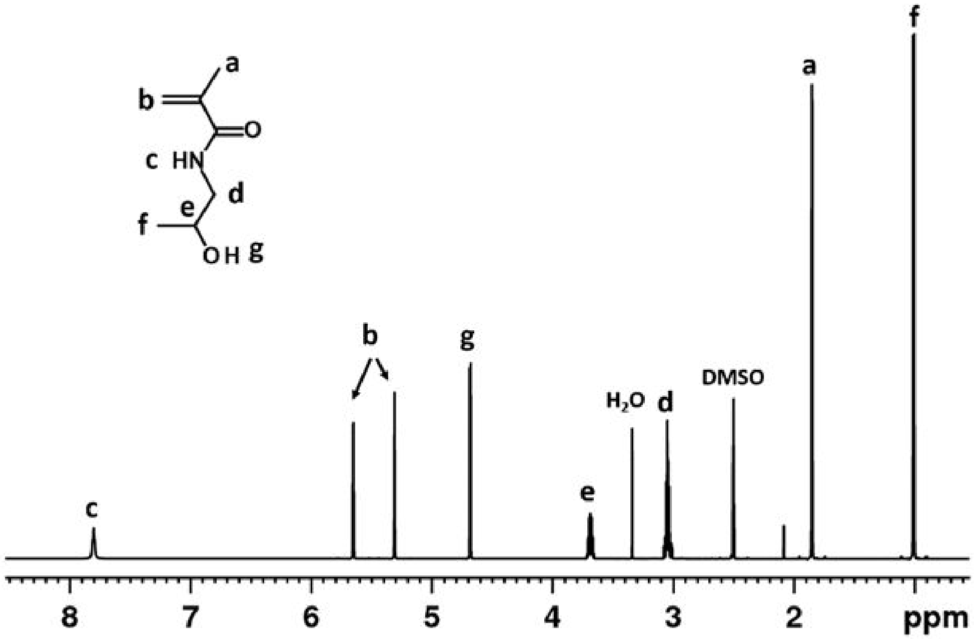

Figure S1. ^{1}H-NMR spectrum of the *N*-(2-hydroxypropyl) methacrylamide (HPMA) in DMSO-d_6.

2) 4-Cyano-4-(((ethylthio)carbonothioyl)thio)pentanoic acid (CEPA) Sodium hydride (60 % in mineral oil, 3.15 g, 0.079 mol) was added to diethyl ether (150 mL) in a 250 mL round bottom flask and was cooled to <10°C. Ethanethiol (4.72 g, 0.076 mol) was added dropwise (over 20 minutes) to the suspension containing sodium hydride and the resultant mixture was allowed to stir for 10 minutes. To this solution, carbon disulfide (6.0 g, 0.079 mol) was then added dropwise. After a further 10 minutes, the resultant ethane trithiocarbonate sodium salt (7.0 g, 0.044 mol) was isolated by filtration by suction using a fritted glass funnel. The ethane trithiocarbonate sodium salt was resuspended in diethyl ether (100 mL) and iodine (5.6 g, 0.022 mol) was added and stirred for 45 minutes at room temperature. Subsequently, the brown suspension was washed two-fold with $Na_2S_2O_3$ (200 mL of 5.0 wt. %) solution and once with brine (200 mL) to yield a yellow ether phase which was collected, dried over $MgSO_4$, filtered and concentrated via rotary evaporation to yield a viscous yellow liquid (3.07 g, 0.011 (mol). ^{1}H-NMR (400 MHz, $CDCl_3$ δ, ppm) 3.30 (q, 4H), 1.34 (t, 6H).

The bis(ethyl trithiocarbonate) was dissolved in ethyl acetate (100 mL) and 4,4'-azobis(4-cyanopentanoic acid) (V-501, 4.57 g, 0.0169 mol) was added. This solution was refluxed under nitrogen at 90-100 °C for 20 hours. The solution was concentrated and purified by flash silica chromatography (30.0 × 5.0 cm) using n-pentane/ethyl acetate/acetic acid (1/1/0.03 v/v/v). After rotary evaporation of the appropriate fractions (Rf = 0.65 in n-pentane/ethyl acetate/acetic acid 1/1/0.03 v/v/v), a viscous yellow liquid was obtained. The yellow liquid was placed at -20 °C, which yielded yellow crystals that were subsequently filtered, washed with ice-cold pentane and dried in vacuo. Yield: 5.76 g.

^{1}H-NMR (400 MHz, $CDCl_3$, δ, ppm): 3.33 (q, 2H, CH_2CH$_3$), 2.67 (m, 2H, CH_2CH$_2$), 2.52 (m, 1H,

CH_2CH_2), 2.39 (m, 1H, CH_2CH_2), 1.86 (s, 3H, CH_3), 1.34 (t, 3H, CH_3). Conversion by ^{1}H-NMR spectroscopy 85%.

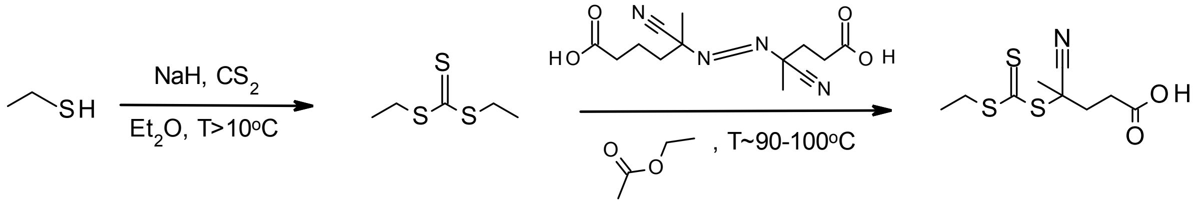

Scheme S2. Schematic representation of the synthesis of 4-cyano-4-(((ethylthio)carbonothioyl)thio)pentanoic acid (CEPA).

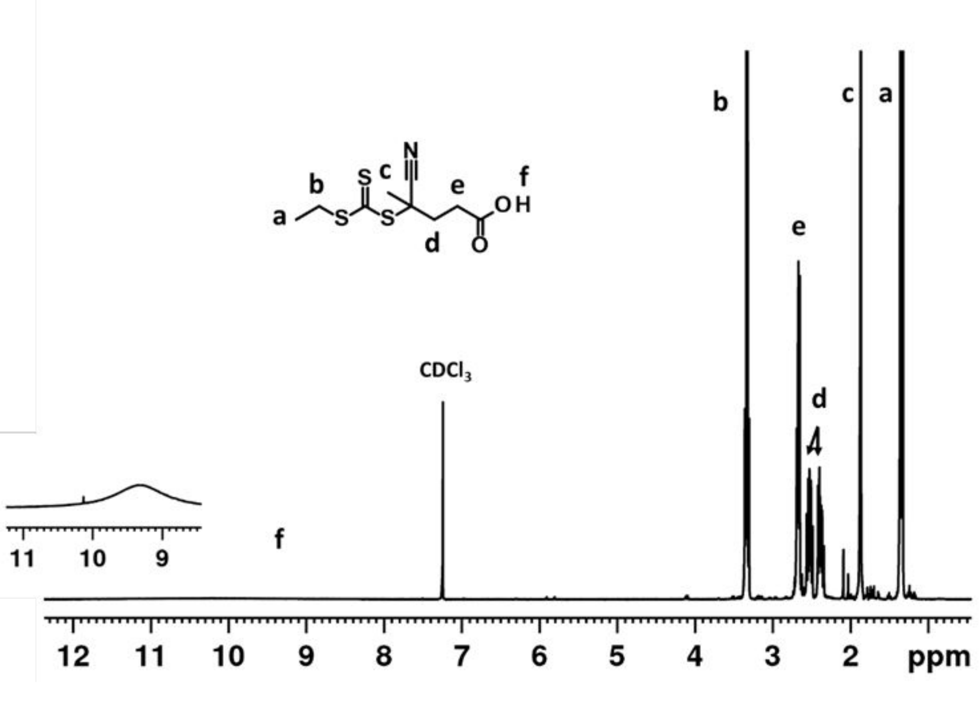

Figure S2. ^{1}H-NMR spectrum of 4-cyano-4-(((ethylthio)carbonothioyl)thio)pentanoic acid in $CDCl_3$.

3) Synthesis of PLA-*b*-PTEGMA diblock copolymer *via* a one-pot/one-step protocol.

A typical protocol for the one-pot/simultaneous ROP of LA and RAFT of triethylene glycol methyl ether methacrylate (TEGMA) is detailed below: LA (390 mg, 2.7 mmol), TEGMA (1.6 mL, 5.0 mmol), CTA-OH (30.0 mg, 0.08 mmol), AIBN (3.2 mg, 0.02 mmol), DMAP (38.0 mg, 0.31 mmol), and 0.75 mL of 1,2-dichloroethane were combined in a dry 10 mL Schlenk flask. The feed ratio used was [LA]:[TEGMA]:[CTA-OH]:[AIBN]:[DMAP]=[35]:[64]:[1]:[0.25]:[0.25]. The reactor was equipped with a magnetic stirrer, sealed, subjected to three freeze-thaw cycles, and then purged with argon gas. The reaction mixture was placed in a preheated oil bath at 75°C. Conversion was determined by comparing the remaining monomer concentration with the initial monomer feed. The final copolymer was purified by dialysis against acetone using a molecular weight cut-off (MWCO) of 2 kDa. The

block copolymer was recovered by evaporating the solvent, dried under vacuum for approximately 24 hours, and then analyzed by ^{1}H-NMR spectroscopy (refer to Figure S3 in ESI) and SEC analysis (refer to Figure S6, black curve). The results are presented in Table 1.

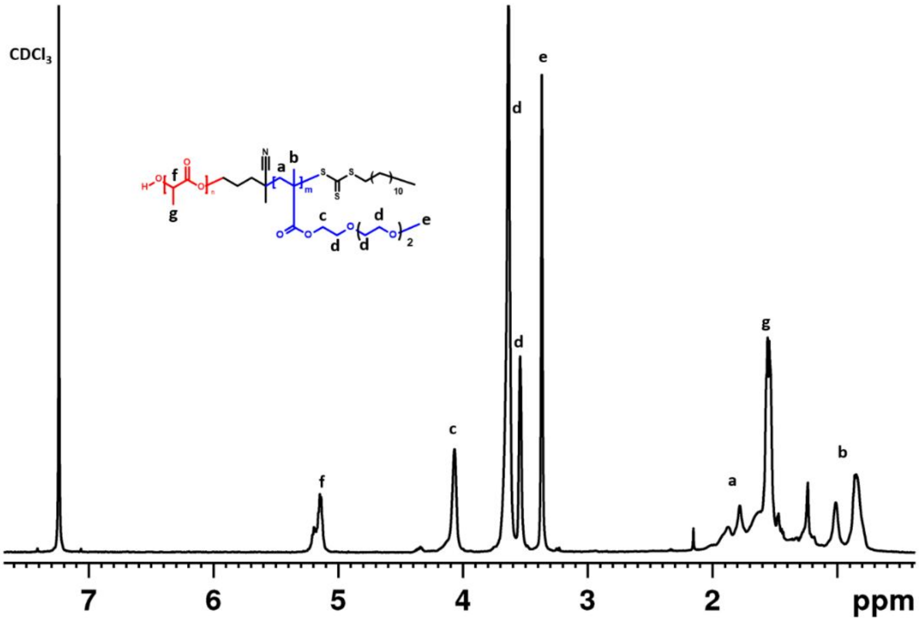

Figure S3. ^{1}H-NMR spectrum of PLA-*b*-PTEGMA diblock copolymer in $CDCl_3$.

4) Synthesis of PHPMA-*g*-PLA graft copolymer *via* one-pot/one-step protocol.

The feed ratio utilized for the one-pot/simultaneous RAFT/ROP of 2-hydroxypropyl methacrylate (HPMA) and LA is as follows: [HPMA]:[LA]:[CEPA]:[AIBN]:[DMAP]=[75]:[35]:[1]:[0.25]:[0.25]. HPMA (1.17 mL, 8.4 mmol), LA (408 mg, 2.8 mmol), CEPA (30.0 mg, 0.081 mmol), AIBN (3.321 mg, 0.020 mmol), DMAP (40.0 mg, 0.32 mmol), and 1.0 mL of methylene chloride, were mixed together in a dry 10 mL Schlenk flask. The graft copolymer was analyzed using ^{1}H-NMR spectroscopy (refer to Figure S4 in ESI) and SEC analysis (refer to Figure S6, red curve). The results are presented in Table 1.

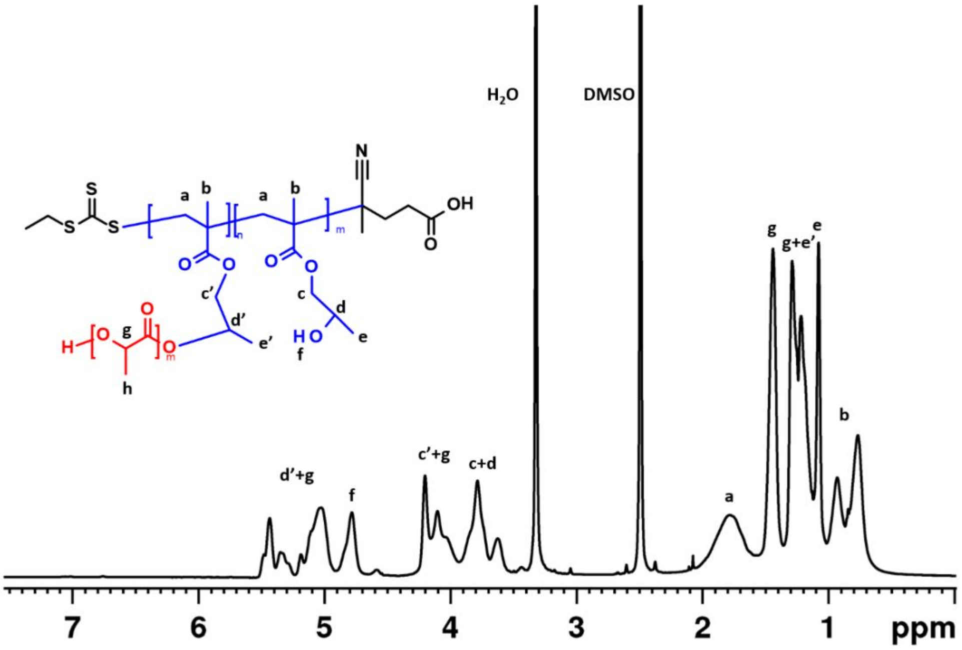

Figure S4. ^{1}H-NMR spectrum of PHPMA-*g*-PLA graft copolymer in DMSO-d$_6$.

5) Synthesis of PHPMAA-*g*-PLA graft copolymer *via* one-pot/one-step protocol.

A typical one-pot/simultaneous RAFT of N-(2-hydroxypropyl) methacrylamide (HPMAA) and ring-opening polymerization (ROP) of LA is described as follows: HPMAA (600 mg, 4.3 mmol), LA (257 mg, 1.79 mmol), CEPA (20 mg, 0.051 mmol), AIBN (2.11 mg, 0.013 mmol), DMAP (25 mg, 0.204 mmol), and 0.75 mL of 1,2-dichloroethane were combined in a dry 10 mL Schlenk flask. The feed ratio used was [HPMA]:[LA]:[CEPA]:[AIBN]:[DMAP]=[84]:[35]:[1]:[0.25]:[0.25]. The synthesis proceeded similarly to the previous two copolymers. Subsequently, the crude product was dissolved in a small amount of 1,4-dioxane, and the graft copolymer was purified using Sephadex LH-20 with 1,4-dioxane as the mobile phase. The copolymer was then precipitated in cold diethyl ether and vacuum-dried to yield a solid. The obtained graft copolymer was characterized using ^{1}H-NMR spectroscopy (see Figure S5 in ESI) and SEC analysis (see Figure S6, green curve). The results are presented in Table 1.

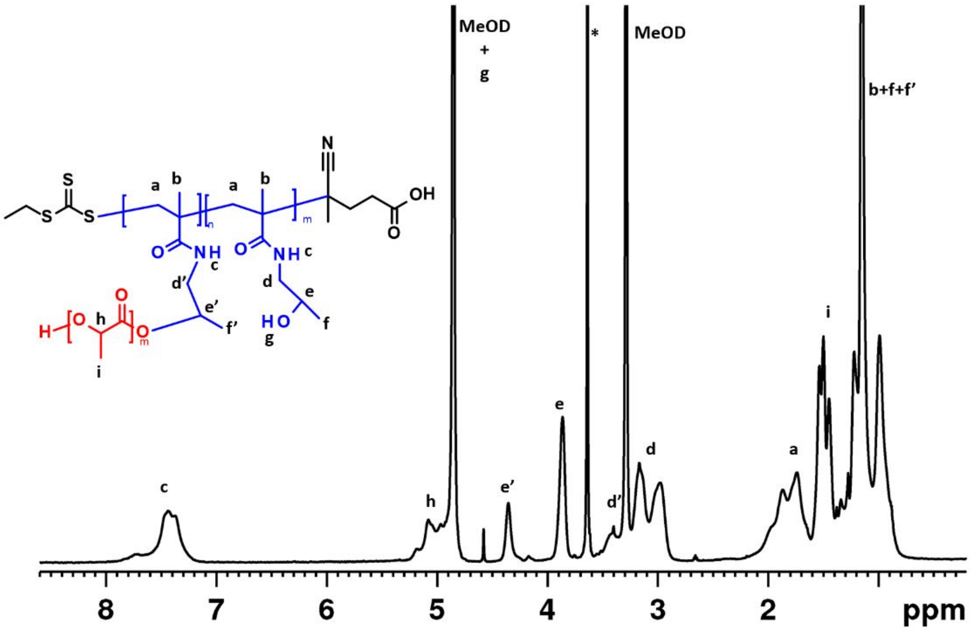

Figure S5. ^{1}H-NMR spectrum of PHPMAA-*g*-PLA graft copolymer in CH_3OD.

Hydrodynamic flow focusing nanoprecipitation microfluidics (MF)

Microfluidics is an emerging field for the production of particles, droplets, capsules and multiple emulsions. Among other common techniques such as nanoprecipitation, film-rehydration and electroformation, microfluidics allows more controlled and precise way for the production of highly monodisperse polymeric structures including micelles, worms and vesicles.

Microfluidics nanoparticle preparation systems with high shear micromixing and hydrodynamic focusing methods give precise particle sizes in the range of ~20 nm to 500 nm. The microfluidics use continuous and controllable laminar flow for the production of high-yield and high-quality nanoparticles. The superior control over the size, shape and morphology of particles enables greater reproducibility and scalability. These substantial improvements in nanoparticle generation are highly valued in pharmaceutical industry compared to conventional batch methods. In this thesis, for production of well-defined nanosized polymersomes was used the commercial microfluidic chip from Dolomite company (Figure S6).

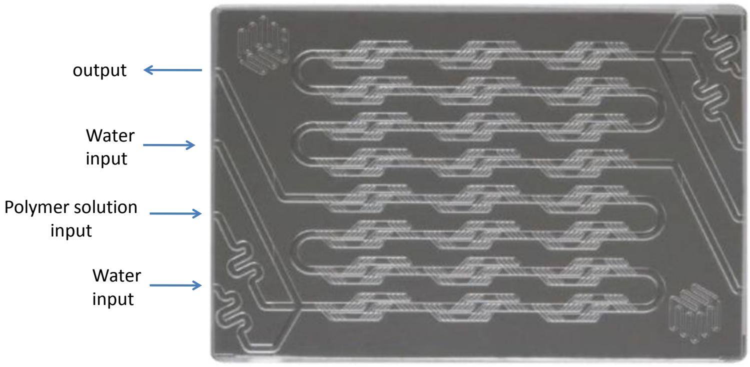

Figure S6. Dolomite setup of the micromixing chip with two water inputs and one polymer solution input.

The mixing time (τ_{mix}) for the hydrodynamic flow focusing using a two-dimensional model is estimated according to equation 1:

$$\tau_{mix} = \frac{w^2}{9D}\frac{1}{(1+1/R)^2} \quad (1)$$

where D is diffusivity of the solvent, w is channel width, and R is the ratio of flow rate of the polymeric stream to the total flow rate of water. For $D \sim 10^{-9}$ $m^2 \cdot s^{-1}$ and $w = 125$ µm equation 1 predicts a mixing time in the range of 0.6 to 7 ms for typical flow ratios ($R = 0.04 - 0.8$) in this device. Particles sizes can be finely tuned by changing the flow ratios between the organic and the aqueous phase, the slowest flow gives the smallest particles which was explained by the decrease in the τ_{mix}. Furthermore, besides the lower particle sizes a decrease in the width of the particle size distribution is observed compared with the bulk nanoprecipitation process. The NPs produced using this microfluidic device always presented size distributions with dispersity below 0.1 when measured by dynamic light scattering.

Characterization techniques:

Size exclusion chromatography (SEC)

The apparent average molecular weights (i.e., number-average molecular weight (M_n) and weight-average molecular weight (M_w) and the molecular weight distribution or dispersity ($Đ$) of the PLA-*b*-PTEGMA, PHPMA-*g*-PLA, and PHPMAA-*g*-PLA copolymers were obtained by SEC performed at 25 ºC with two PLgel MIXED-C columns (300 x 7.5 mm, SDV gel with particle size 5 µm; Polymer Laboratories, USA) and with UV (UVD 305; Watrex, Czech Republic) and RI (RI-101; Shodex, Japan) detectors. N,N-Dimethylformamide (Sigma Aldrich, Czech Republic) with LiBr (0.01 % v/v) was used as mobile phase at flow rate of 1 mL min^{-1}. The M_n values were calculated using the Clarity software (Dataapex, Czech Republic). Calibration with poly(methyl methacrylate) (PMMA) standards was used.

The dynamic light scattering (DLS)

The DLS measurements were performed using an ALV CGE laser goniometer consisting of a 22 mW HeNe linear polarized laser operating at a wavelength (λ = 632.8 nm), an ALV 6010 correlator, and a pair of avalanche photodiodes operating in pseudo-cross-correlation mode. The samples were loaded into 10 mm diameter glass cells and maintained at 37 ± 1 °C. The data were collected using the ALV Correlator Control software and the counting time was 45 s. The measured intensity correlation functions $g_2(t)$ were analyzed using the algorithm REPES (incorporated in the GENDIST program)[57], resulting in the distributions of relaxation times shown in equal area representation as $\tau A(\tau)$. The mean relaxation time or relaxation frequency ($\Gamma = \tau^{-1}$) is related to the diffusion coefficient (D) of the nanoparticles as $D = \frac{\Gamma}{q^2}$ where

$q = \frac{4\pi n \sin\frac{\theta}{2}}{\lambda}$ is the scattering vector being n the refractive index of the solvent and θ the scattering angle. The hydrodynamic radius (R_H) or the distributions of R_H were calculated by using the Stokes-Einstein relation:

$$\boldsymbol{R_H = \frac{k_B T q^2}{6\pi\eta D}\tau} \quad (2)$$

being k_B the Boltzmann constant, T the absolute temperature, and η the viscosity of the solvent. The transition temperature (T_{tr}), characterizing the polymer chain conformation changes, was evaluated from the temperature dependence of the hydrodynamic diameter (D_H) and was determined from the intersection point of two lines formed by the linear regression of a lower horizontal asymptote and a vertical section of the S-shaped curve (sigmoidal curve) fit.

The small-angle X-ray scattering (SAXS)

The SAXS experiments were recorded by using a pinhole camera (MolMet, Rigaku, Japan, modified by SAXSLAB/Xenocs) attached to a microfocused X-ray beam generator (Rigaku MicroMax 003) operating at 50 kV and 0.6 mA (30 W). The samples were sealed into boro-silicate capillaries (~2 mm diameter) and the scattering intensities were recorded by using a Pilatus 300 K detector with an exposure time of 480 min. The scattering profiles were fitted by using the SASfit software. The PLA-*b*-PTEGMA and PHPMA-*g*-PLA nanoparticles were fitted by the core-shell model (with the core representing the hydrophobic PLA component and shell representing the amphiphilic component of the graft copolymer). Polydispersity of the particles was accounted for in the form factors by using the log-normal distribution. The PHPMAA-*g*-PLA worms were fitted by the combination of the WormLikeChainEXV model (to describe the worm-like chains) and the extended Guinier law model (to describe the spherical PHPMAA corona block). The effect of solvent (water) was approximated by the constant function $I_{H2O} = 3.41$.

In the core-shell model, the scattering intensity is defined by equation 3:

$$I(q) = [K(q, R_{core}, \Delta\eta_{core}) + K(q, R_{core} + \Delta R_{shell}, \Delta\eta_{shell}) - K(q, R_{core}, \Delta\eta_{shell} - \Delta\eta_{core})]^2 + \text{background} \quad (3)$$

with

$$\boldsymbol{K(q, R, \Delta\eta) = \frac{4}{3}\pi R^3 \Delta\eta 3 \frac{\sin(qR) - qR\cos(qR)}{(qR)^3}} \quad (4)$$

where R_{core} is the radius of the core, ΔR_{shell} is the shell thickness, and $\Delta\eta$ is the scattering length density difference between core/shell and the solvent.

The log-normal distribution is defined by equation 5:

$$f(x) = \frac{1}{Norm}\frac{1}{x\sigma}exp\left(-\frac{1}{2}\left(\frac{ln(x)-\mu}{\sigma}\right)^2\right) \quad (5)$$

where Norm is a normalization factor determined during numerical calculation, μ=ln(x_{med}), x_{med} is the median value of the distribution and σ (=PD or polydispersity ratio) is a parameter describing the width of the underlying normal distribution. The values of these parameters obtained by fitting for PLA-*b*-PTEGMA and PHPMA-*g*-PLA NPs from this work are summarized in Table S1.

Table S1. Parameters of the core-shell model for PLA-*b*-PTEGMA and PHPMA-*g*-PLA NPs at various temperatures with a log-normal distribution of core and shell radii to account for the polydispersity of the samples.

polymer	PLA-*b*-PTEGMA					PHPMA-*g*-PLA		
T [°C]	16	30	45	55	60	5	25	60
r_{core} [nm]	30.1	31.5	32.0	33.4	17.0	58.1	57.7	59.2
PD (r_{core})	0.38	0.39	0.42	0.49	0.50	0.26	0.28	0.36
$\Delta\eta_{\text{core}}$[a] [Å^{-2}]	2.3E-5	2.4E-5	2.6E-5	2.9E-5	1.3E-4	6.6E-5	6.5E-5	1.8E-5
r_{shell} [nm]	4.4	3.8	4.6	6.3	50.4	35.7	41.7	49.3
PD (r_{shell})	0	0	0	0	0.27	0.24	0.25	0.47
$\Delta\eta_{\text{shell}}$[a] [Å^{-2}]	7.1E-5	8.0E-5	7.0E-5	6.6E-5	-8.0E-6	5.6E-5	5.5E-5	4.8E-5

[a] scattering length density of water was fixed at 1E-4 Å^{-2}.

The WormLikeChainEXV model is characterized by the structure factor $F_S(q,R,L)$ which consists of a cross-section term $F_{cs}(q,R)$ and a longitudinal term $F_L(q,L)$, so that $F_S = F_{cs}$□F_L with

$$F_{cs}(q,R) = \left[\frac{2B_1(qR)}{qR}\right]^2 \quad (6)$$

where $B_1(x)$ is the first order Bessel function of the first kind and

$$F_L(q,L) = 2Si(qL)\ /\ (qL) - 4sin^2(qL\ /\ 2)\ /\ \left(q^2L^2\right) \quad (7)$$

where

$$Si(x) = \int_0^x t^{-1} sin\, t\, dt. \quad (8)$$

The parameter R describes the cross-section radius of the worm, L is the total length of the worm and b is the Kuhn length which describes the stiffness of the chain and corresponds to twice the length of locally stiff segments. The exponents of the Bessel function are strongly dependent on the L/b ratio and the behavior is quite complex, so we refer the reader to the original text (reference 72 in the main manusript) for details.

The extended Guinier law model defines scattering intensity as

$$I(q) = \left\{\frac{1 \quad \text{for } \alpha = 0}{\alpha\pi q^{-\alpha} \text{ for } \alpha = 1{,}2}\right\} A exp\left(-\frac{R_g^2 q^2}{3-\alpha}\right) \quad (9)$$

where Rg is the radius of gyration and α is the dimensionality factor which is 0 for a spheroid, 1 for a rod-like shape and 2 for a plane. The parameter obtained by fitting for PHPMAA-*g*-PLA worms from this work are summarized in Table S2.

Table S2. Parameters of the WormLikeChainEXV and extended Guinier law models PHPMAA-*g*-PLA worms.

WormlikeChainEXV		extended Guinier law	
R [nm]	13	R_g [nm]	1736
L [nm]	5592	α	0
b [nm]	870		

The transmission electron microscopy (TEM)

Observations were studied using a transmission electron microscope, specifically the Tecnai G2 Spirit Twin 120kV (FEI, Czech Republic), operated at an acceleration voltage of 120kV. For the procedure, 2 µL of the aqueous solutions were dropped onto a copper TEM grid (400 mesh) coated with a thin, electron-transparent carbon film. Excess solution was removed by touching the bottom of the grid with filtering paper. This rapid removal of the solution occurred after 1 minute of sedimentation to minimize oversaturation during the drying process. Nanoparticles were negatively stained with uranyl acetate (2 µL of 2 wt.% solution dropped onto a not completely dried grid and removed after 30 s using the same method described above). The samples were then left to dry completely at ambient temperature and subsequently observed in the TEM microscope. Under these conditions, the micrographs displayed a negatively stained background with bright nanoparticles.

Before sample preparation at different temperatures, solutions and microscopic grids were tempered for 24 hours either in a refrigerator (5°C) or in an oven (60°C). Approximately 2.0 μL of the tempered aqueous solution was directly applied to the tempered microscopic grid, following the same procedure as described above, either in the refrigerator or in the oven.

After complete drying, the particles were negatively stained at room temperature with uranyl acetate, following the procedure described above.

Isothermal titration calorimetry (ITC)

The nanoparticles, from linear PLA-*b*-PTEGMA, PHPMA-*g*-PLA and PHPMAA-*g*-PLA graft copolymers 5.0 mg mL^{-1} each, in ultrapure water were titrated with human blood plasma, diluted with PBS to 10% vol/vol on ITC 200 titration calorimeter (Malvern Panalytical, Malvern, UK) at 20, 37 and 54°C. The ultrapure water was selected as a dispersion medium for polymer particles because the undiluted PBS triggered the gelation of the particles. Diluted human plasma was added in 20 consecutive injections, the first injection was always 0.4 μL followed by 19 injections of 2.0 μL. Additionally, the diluted plasma was titrated similarly to ultrapure water at all experimental temperatures to account for its heat of dilution. The calorimetry data were plotted as heat per injection, normalized to the injection volume (μJ/μL) vs the ratio of the injected volume of diluted human blood plasma (cumulative) to the mass of particles (μL/mg).

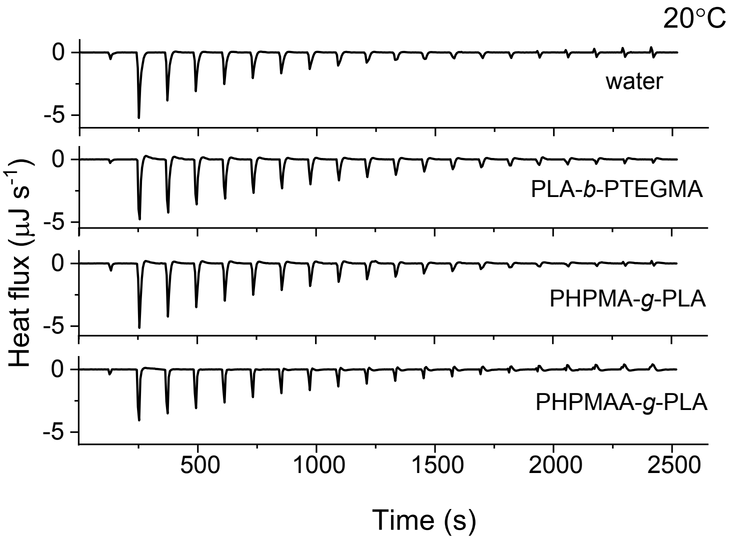

Figure S7. The ITC titration involved diluting human blood plasma (10% vol/vol PBS) with ultrapure water, and conducting the process with PLA-*b*-PTEGMA, PHPMA-*g*-PLA, and PHPMAA-*g*-PLA nanoparticles at a concentration of 5.0 mg mL^{-1} at 20 °C.

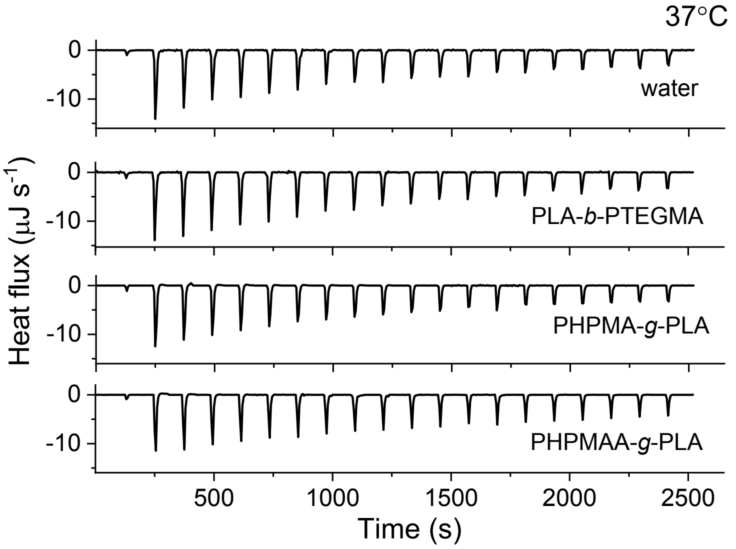

Figure S8. The ITC titration involved diluting human blood plasma (10% vol/vol PBS) with ultrapure water, and conducting the process with PLA-*b*-PTEGMA, PHPMA-*g*-PLA, and PHPMAA-*g*-PLA nanoparticles at a concentration of 5.0 mg mL^{-1} at 37°C.

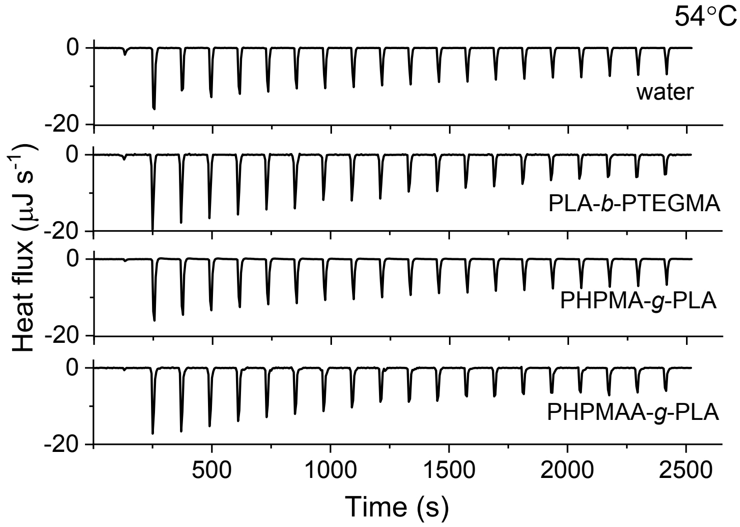

Figure S9. The ITC titration involved diluting human blood plasma (10% vol/vol PBS) with ultrapure water, and conducting the process with PLA-*b*-PTEGMA, PHPMA-*g*-PLA, and PHPMAA-*g*-PLA nanoparticles at a concentration of 5.0 mg mL^{-1} at 54°C.
